# Styrene-Assisted Maleic Anhydride Grafted Poly(lactic acid) as an Effective Compatibilizer for Wood Flour/Poly(lactic acid) Bio-Composites

**DOI:** 10.3390/polym9110623

**Published:** 2017-11-15

**Authors:** Jun Du, Youyong Wang, Xinfeng Xie, Min Xu, Yongming Song

**Affiliations:** 1 Key Laboratory of Bio-Based Material Science and Technology (Ministry of Education), Material Science and Engineering College, Northeast Forestry University, Harbin 150040, China; dujun9402@163.com (J.D.); wangyouyong2018@163.com (Y.W.); xumin1963@126.com (M.X.); 2 School of Forest Resources and Environmental Science, Michigan Technological University, Houghton, MI 49931, USA; xinfengx@mtu.edu

**Keywords:** maleic anhydride grafted poly(lactic acid), styrene, wood flour, poly(lactic acid), bio-composites, interfacial adhesion

## Abstract

This study aimed to evaluate the effect of styrene-assisted maleic anhydride-grafted poly(lactic acid) (PLA-*g*-St/MAH) on the interfacial properties of wood flour/poly(lactic acid) (PLA) bio-composites. PLA-*g*-St/MAH was synthesized by free-radical melt grafting using styrene as a comonomer and dicumyl peroxide as an initiator. The structure of PLA-*g*-St/MAH was characterized by Fourier transform infrared spectroscopy. Wood flour/PLA composites were prepared by compression molding using PLA-*g*-St/MAH as a compatibilizer. The effects of PLA-*g*-St/MAH on the rheological and mechanical properties, as well as on the fractured surface morphology of the composites were investigated. Results indicated that storage modulus, complex viscosity, equilibrium torque, and shear heat were significantly increased. The mechanical properties of the wood flour/PLA composites were also significantly increased after the addition of PLA-*g*-St/MAH. The maximum values were achieved at the loading rate of 3 wt % because of the improved interfacial adhesion between the wood flour and the PLA matrix.

## 1. Introduction

Wood plastic composite (WPC), an environmentally friendly material, has drawn increasing interest in recent years, owing to the distinct combination of high strength and elasticity derived from wood fiber or natural fibers, as well as durability and fatigue resistance derived from its polymer matrix [1,2,3]. WPC products have many applications in the construction, interior decorating, household appliance, and transportation industries [4]. With growing awareness of the global environmental energy crisis and resource constraints, extensive research has been conducted into the use of bio-based and biodegradable plastics, such as poly(lactic acid) (PLA) [5,6], poly(butylene succinate) [7], and poly(butylene adipate-*co*-terephthalate) [8], in WPC to replace non-degradable plastics, such as polyethylene, polypropylene, polyvinyl chloride, and polystyrene, which are derived from petroleum resources. The use of biodegradable polymers to produce environmentally-friendly bio-composites is a promising trend [9,10].

PLA is a non-toxic and biodegradable bio-based thermoplastic polyester with numerous desirable properties, such as high strength and high stiffness [11,12]. It is a polyester resulting from the polymerization of lactic acid, a bio-based monomer produced by the fermentation of biomass feedstocks. However, extensive application of PLA has not yet been achieved because of certain limitations, such as brittleness, slow crystallization, and relatively high cost [13]. To overcome these disadvantages, low-cost natural fibers with higher modulus and rigidity, such as wood flour [14], have been considered as a reinforcement for PLA. Wood flour/PLA bio-composites can not only reduce the overall cost of the material and enhance the brittleness of PLA, but are also potentially completely biodegradable [15,16].

However, the incompatibility between PLA and wood fiber (hydrophobicity versus hydrophilicity) leads to poor wettability and interfacial adhesion between the two materials [17]. Therefore, interfacial modification between wood fiber and PLA matrix is the key to improving the mechanical properties of wood flour/PLA bio-composites. To improve the compatibility of these two phases in the bio-composites, many modification methods have been developed, including alkali treatment [18,19], silane modification [20,21], acetylation [22,23], addition of compatibilizers [24,25], etc. Recently, one of the more promising interfacial modification approaches was adopted for use in binary/ternary incompatible PLA blends or composites, which included grafting a reactive moiety, such as maleic anhydride (MAH), onto the polymer matrix and then using this graft copolymer functioning as a compatibilizer in immiscible systems [26,27]. MAH grafted PLA has been used as a compatibilizer in PLA blends with starch [28,29], clay [30], and natural fibers [31,32] to improve the interfacial adhesion.

Graft copolymers were also used in WPC, and relatively higher grafting degrees of the graft copolymer were found to be beneficial to improving the compatibility between the wood flour and the PLA matrix [16,33,34]. The addition of styrene (St) was shown to be an effective way of increasing the grafting degree of MAH onto a polymer matrix, and could activate MAH, as well as restrain the degradation of the polymer matrix [35]. The presence of St as a comonomer donates electrons, which promote the activity of MAH, helping to render its unsymmetrical structure and its π radical-anion bonds [36]. Although styrene has been reported to be a comonomer for polyolefin-*g*-St/MAH systems, less focus has been directed toward St-assisted MAH-grafted polyesters, such as PLA. Moreover, no papers have reported on the preparation of wood flour/PLA bio-composites with styrene-assisted maleic anhydride-grafted poly(lactic acid) (PLA-*g*-St/MAH) as a compatibilizer. The goal of this study was to explore the effect of PLA-*g*-St/MAH on the interfacial properties of wood flour/PLA bio-composites.

In the current study, a novel PLA-*g*-St/MAH was synthesized using St as a comonomer and dicumyl peroxide (DCP) as an initiator by melting graft reactions. This is the first attempt to incorporate PLA-*g*-St/MAH into wood flour/PLA bio-composites to improve interfacial adhesion between the wood flour and the PLA matrix. In addition to the effects of PLA-*g*-St/MAH on mechanical properties and morphology, the rheological properties of the composites with the addition of PLA-*g*-St/MAH were examined. The correlation between the rheological behavior and the mechanical properties was further clarified, as well.

## 2. Materials and Methods

### 2.1. Materials

PLA (3001D, average molecular weight of 72,000 g/mol) was provided by NatureWorks (Minnetonka, MN, USA) with a density of 1.24 g·cm^−3^ and a melt flow index of 13.6 g/10 min (190 °C, 2.16 kg according to ASTM D1238). Wood flour with a mesh size of 100 was supplied by Sanqi Wood Flour Agency (Linyi, China). MAH was provided by Tianjin Guangfu Chemical Reagent (Tianjin, China). Styrene was purchased from Tianjin Fuchen Chemical Reagent (Tianjin, China). DCP was provided by Shanghai Macklin Biochemical Co., Ltd. (Shanghai, China). Polyethylene Wax (PE wax) was supplied by Shandong Qilu Petrochemical Engineering Co., Ltd. (Zibo, China) and used as the lubricant in the preparation of wood flour/PLA composites.

### 2.2. Preparation of Samples

#### 2.2.1. PLA-*g*-St/MAH

PLA was dried in a vacuum oven at 50 °C for 12 h before use. MAH, St and DCP (4.5, 9 and 0.45% based on the weight of neat PLA) were first dissolved in 5–10 mL acetone and then sprayed onto the surface of PLA pellets to obtain a uniform distribution of chemicals in the mixtures. After complete evaporation of the solvent, the mixtures were fed into a torque rheometer (HAAKE PolyLab OS, Thermo Electron, Karlsruhe, Germany) to obtain the PLA-*g*-St/MAH, where melting graft reactions occurred at 180 °C and a fixed screw speed of 50 r/min for 8 min. The graft copolymers were cooled, granulated, and dried for further use. For comparison, neat PLA and PLA-*g*-MAH were also processed under the same conditions.

#### 2.2.2. Wood Flour/PLA Composites

Wood flour was dried for 12 h at 103 °C, and PLA was dried for 12 h at 50 °C in a vacuum oven prior to composite fabrication. The wood flour, PLA-*g*-St/MAH, and PLA resin were premixed in a high-speed blender at room temperature for 10 min. PE wax was then added to the mixture and blended for another 5 min. Then, the mixtures were transferred to a twin-screw extruder (SJSH-30, Nanjing Xiangsu Machinery, Nanjing, China). The temperature of the extruder barrel was set at 155/160/170/180/180/170/165 °C (from the feeder zone to the die), and the rotation speed was set to 30 rpm. Extrudate pellets were molded into composite sheets in a plastic compression molding press (SL-6, Harbin Special Plastic Products, Harbin, China) at 180 °C with a constant pressure of 10 MPa. Pressed wood flour/PLA composite samples were then cut into standard specimens for the following test. Compositions of various wood flour/PLA composites are presented in Table 1.

### 2.3. Characterizations

#### 2.3.1. Fourier Transform Infrared (FT-IR) Spectroscopy

To obtain a pure graft copolymer, approximately 2.5 g of the processed PLA-*g*-St/MAH sample was dissolved in 150 mL chloroform using reflux, followed with precipitation in excessive ethanol after cooling. The precipitates were filtered out and then washed three times with fresh ethanol to remove unreacted monomers, and then dried in the vacuum oven at 50 °C for 12 h. The purified graft copolymers were characterized using an FT-IR spectrometer (Thermo Nicolet 6700, Thermofisher, Waltham, MA, USA). The FT-IR spectra were recorded in the range of 600–4000 cm^−1^.

#### 2.3.2. Rotational Rheometry

Dynamic rheological tests of the wood flour/PLA composites were conducted using a rotational rheometer (AR2000ex, TA Instruments, New Castle, DE, USA) equipped with 25 mm ETC parallel plate geometry (diameter = 25 mm, gap = 2000 μm) at a temperature of 180 °C. Dynamic oscillatory frequency sweeps were conducted by applying 0.1% strain within the linear viscoelastic region, where rheological properties are not strain dependent, over a frequency range from 628.3 to 0.06283 rad/s.

#### 2.3.3. Torque Rheometry

The torque rheological properties of the wood flour/PLA composites were characterized on a torque rheometer (HAAKE PolyLab OS, Thermo Electron, Karlsruhe, Germany) with roller rotors. Extruded pellets were tested at 180 °C with a rotor speed of 50 r/min for 10 min and a constant filling degree of 75%. The average values of the torque and temperature during the last 2 min of the test were used as the equilibrium torque and temperature of the sample.

#### 2.3.4. Mechanical Property Tests

The tensile and flexural properties of the composites were tested on an electronic universal mechanical testing machine (CMT5504, MTS Systems, Shenzhen, China) using a crosshead speed of 5 mm/min in accordance with ASTM D638-2014 and ASTM D790-2010, respectively. The impact property of the composites was tested using an impact testing machine (XJ-50G, Chengde Dahua Testing Machine, Chengde, China) in accordance with GB/T 1043.1-2008. Eight replicate specimens of each sample were tested. Statistical analysis of the results was conducted with a significance level of 0.05.

#### 2.3.5. Scanning Electron Microscopy (SEM)

The fracture surface morphologies of the wood flour/PLA composites were studied using a scanning electron microscope (Quanta 200, FEI, Hillsboro, OR, USA) with an acceleration voltage of 15 kV. The samples were cooled in liquid nitrogen to produce brittle failure, and flat fracture surfaces of the samples were obtained. The fracture surfaces were then sputter-coated with gold before being loaded into the observation chamber of the SEM.

## 3. Results and Discussion

### 3.1. Characterization of PLA-g-St/MAH

**Figure 1:**
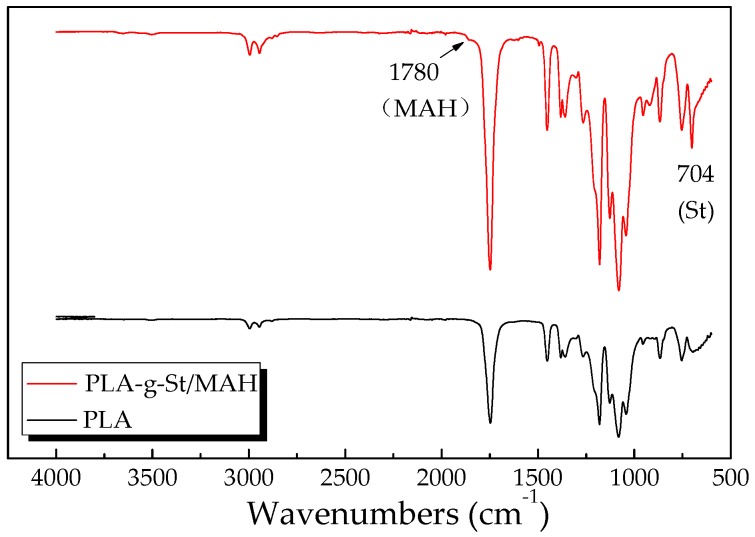
The FT-IR spectrum of PLA and PLA-*g*-St/MAH.

Figure 1 shows the FT-IR absorbance spectra of neat PLA and PLA-*g*-St/MAH in the range of 600–4000 cm^−1^. As shown in Figure 1, the PLA shows strong absorption peaks of ether bond (C–O stretching) at 1180, 1130 and 1083 cm^−1^. The peak at 1746 cm^−1^ is assigned to the C=O stretching of the carboxyl groups of PLA. This peak at 1746 cm^−1^ was significantly strengthened for the PLA-*g*-St/MAH sample because of the addition of MAH, which contains carbonyl bonds (C=O) [37]. The FT-IR spectrum of the PLA-*g*-St/MAH sample also shows an extra shoulder at 1780 cm^−1^ that is not present in that of the PLA sample. This peak is assigned to the ester carbonyl stretching vibration of the copolymer. Similar results have been reported previously by Yu et al. [38]. The characteristic absorption peak of a C–H bond near 3000 cm^−1^ appears to exhibit a slightly increased intensity.

**Figure 2:**
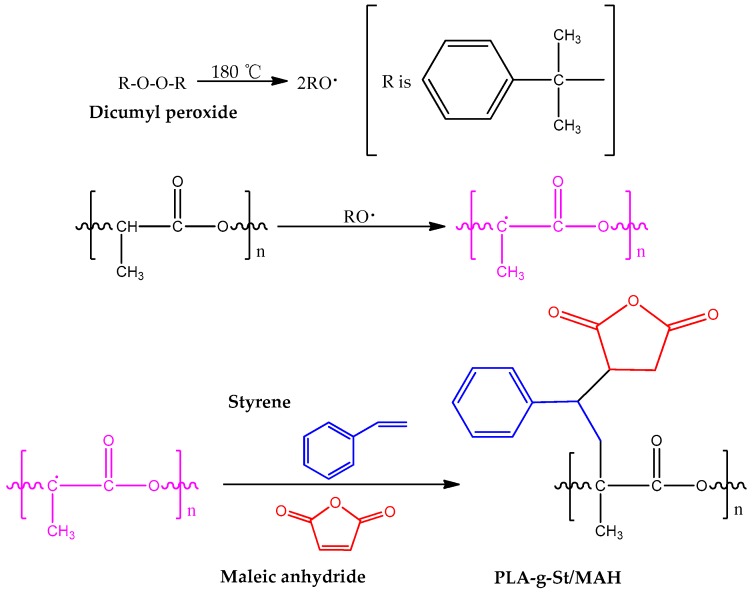
Possible mechanism for melt grafting of MAH onto PLA with St as the comonomer.

In addition, the PLA-*g*-St/MAH sample showed a peak at 704 cm^−1^ which is assigned to the asymmetric stretching vibration of the phenyl group in styrene [35,39]. No peak of C=C (1630 cm^−1^) was observed in either PLA or PLA-*g*-St/MAH. These results indicate that the unreacted MAH and St monomer were completely removed during purification. These results also confirmed that the graft copolymer of PLA-*g*-St/MAH was obtained by free-radical melt grafting with St as the comonomer and DCP as the initiator. The possible mechanism of the grafting reaction [36,40] is shown in Figure 2.

### 3.2. Dynamic Rheological Properties

**Figure 3:**
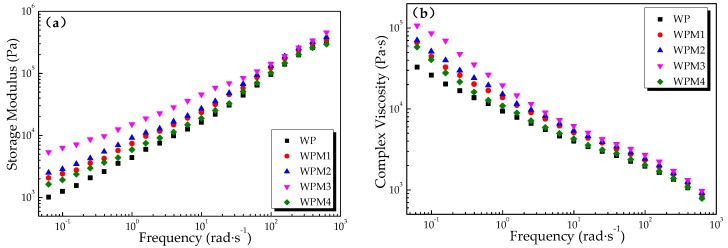
Dynamic rheological properties of the wood flour/PLA composites with different PLA-*g*-St/MAH loading rates: (**a**) storage modulus versus frequency and (**b**) complex viscosity versus frequency.

Rotational rheometry is the method commonly used to characterize the effects of additives on the melt flow behavior of polymer composites. It is sensitive to the molecular structural differences and interfacial interactions of different phases in a composite [41]. Figure 3 shows (a) the storage modulus (*G'*) and (b) complex viscosity (η*) of the wood flour/PLA composites with different loading rates of PLA-*g*-St/MAH during dynamic oscillatory frequency sweeps by the rotational rheometer.

As shown in Figure 3a, the storage modulus of the wood flour/PLA composite was increased with an increase in frequency. In the low-frequency region, where the flow behavior is influenced more by the wood filler particles than the PLA matrix, *G'* increased markedly with the increase of the PLA-*g*-St/MAH loading rates, indicating that the elasticity of the wood flour/PLA composite melt was improved. The reason for this is that the interaction between the wood flour and the PLA matrix was enhanced with the addition of PLA-*g*-St/MAH, thus improving the compatibility between the wood flour and PLA.

As observed, the complex viscosity of the wood flour/PLA composite exhibited a downward trend with an increase in frequency, as shown in Figure 3b, which is a typical occurrence of the shear thinning behavior of the PLA matrix in the composite, owing to insufficient time for the PLA chains to rebuild the original wood filler particle distribution. Compared with the WP sample without the compatibilizer, the addition of PLA-*g*-St/MAH increased the η* of the composite melts. This increase was mainly attributed to the reaction of anhydride groups from the PLA-*g*-St/MAH with the hydroxyl groups from the wood flour surface to form ester or hydrogen bonds, increasing the interface adhesive effect of the wood flour and the PLA matrix. The improved interfacial adhesion between the wood flour and the PLA matrix results in higher resistance to flow. Intermolecular forces of the wood flour/PLA composite melts were simultaneously enhanced with the addition of PLA-*g*-St/MAH, which increased the resistant of the composite melt structure to the alternating stress. These all improved the η* of the wood flour/PLA composites. In addition, the η* of the composite melt with 3 wt % PLA-*g*-St/MAH content was the highest, and the lubricant effect [41,42] of PLA-*g*-St/MAH could explain the reduction in the *G'* and η* of the composite when the content exceeds 3 wt %.

### 3.3. Torque Rheometry

**Figure 4:**
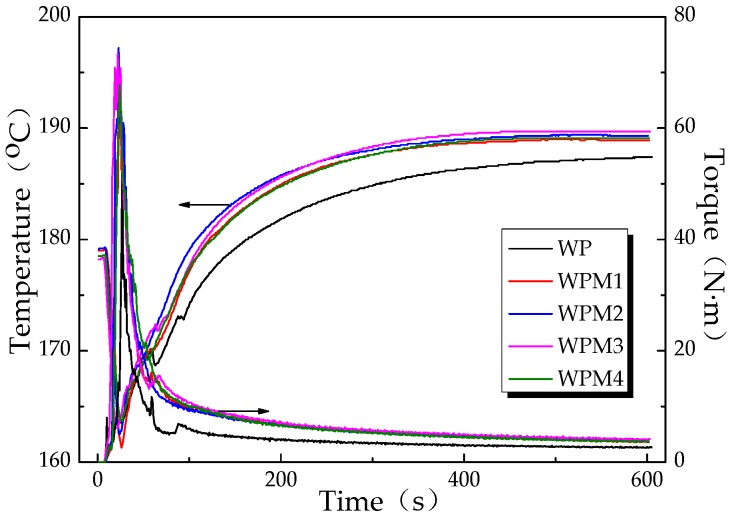
Curves depicting torque and temperature versus time of wood flour/PLA composites with different PLA-*g*-St/MAH loading rates.

Changes in torque and temperature versus time during the torque rheological testing of the wood flour/PLA composite are shown in Figure 4. The specific statistics of the equilibrium torque (*T*_e_) and the shear heat (Δ*T*) of the composites are listed in Table 2.

As shown in Figure 4, a rapid increase in torque occurred when the extruded pellets were added to the mixing chamber at the beginning of the tests. The reason for this was that the cold solid pellets hindered the free rotation of the rotors. As the pellets were heated and began to melt, the torque gradually dropped, owing to the combined action of heating and shearing of the rotors. The equilibrium torque, which reflects the apparent viscosity and flow properties of the molten system [43], was reached when the materials were blended evenly. The smaller the equilibrium torque, the better the flowability of the composite system. The results in Table 2 show that the *T*_e_ value of the composite system increased after PLA-*g*-St/MAH was added, and that the WPS3 sample obtained the highest *T*_e_. This finding matched the results of the complex viscosity of the composite previously described.

Figure 4 also shows that the temperature of the composite melts decreased first, owing to the addition of solid materials, and then increased gradually as a result of the heat produced by shear friction. Δ*T*, the difference between the equilibrium temperature and the initial temperature, indicates the shear heat produced during the material process. Table 2 indicates that Δ*T* of the composite melt increased after the addition of PLA-*g*-St/MAH. Among them, the *T*_e_ and Δ*T* of the WPS3 composite sample were the highest, which further confirmed that PLA-*g*-St/MAH significantly improves the interfacial bonding between the wood flour and the PLA matrix with the addition of 3 wt %. Consequently, the shear resistance and shear heat of the wood flour/PLA composites increased significantly.

### 3.4. Mechanical Properties

**Figure 5:**
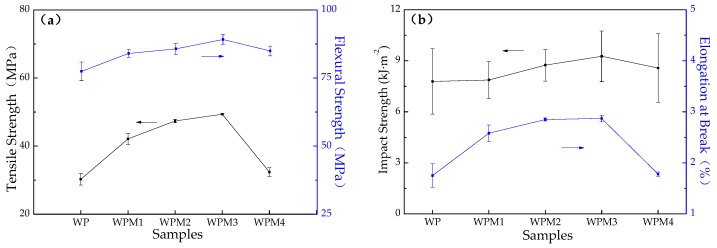
Mechanical properties of wood flour/PLA composites with different PLA-*g*-St/MAH loading rates: (**a**) tensile strength and flexural strength; (**b**) impact strength and elongation at break.

Figure 5 illustrates the mechanical properties of wood flour/PLA composites with different loading rates of PLA-*g*-St/MAH. As shown in Figure 5, both tensile strength and flexural strength of the wood flour/PLA composite were enhanced effectively, and reached a maximum at 3 wt % of the PLA-*g*-St/MAH content. Compared with the WP sample without the addition of PLA-*g*-St/MAH, the WPS3 sample with 3 wt % PLA-*g*-St/MAH content exhibited a 63.06% increase in tensile strength, 15.09% increase in flexural strength, 19.02% increase in impact strength, and 64.00% increase in elongation at break.

**Figure 6:**
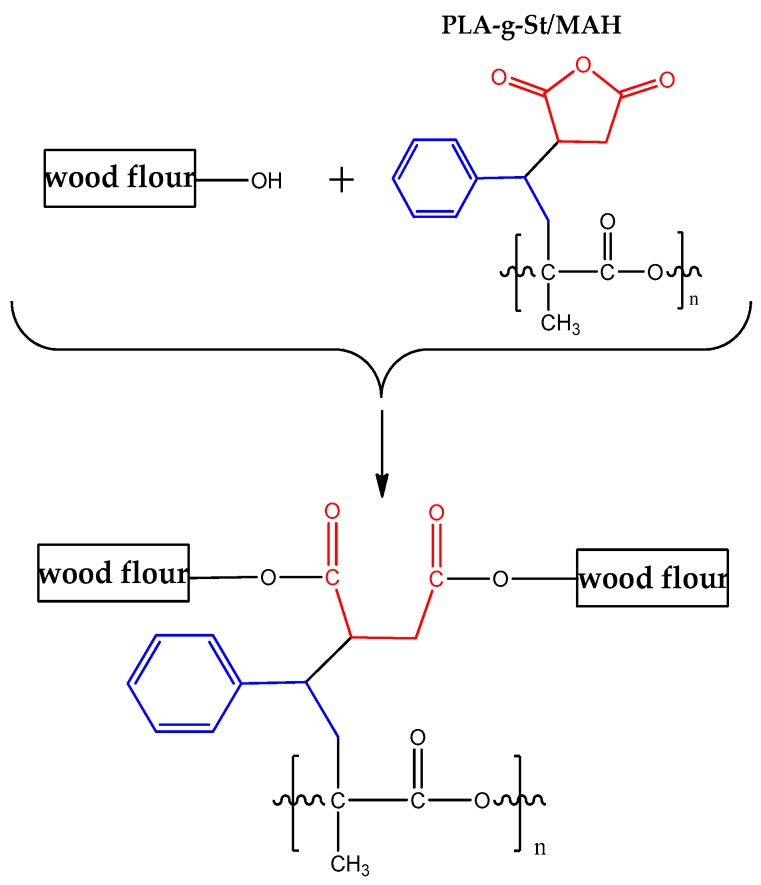
Possible reaction mechanism of wood flour/PLA composites in the presence of PLA-*g*-St/MAH.

The wood flour/PLA composites with PLA-*g*-St/MAH showing higher mechanical properties than that without PLA-*g*-St/MAH proves that PLA-*g*-St/MAH is able to significantly improve the mechanical properties of wood flour/PLA composites. On one hand, PLA-*g*-St/MAH exhibited good compatibility with the PLA matrix of the wood flour/PLA composites, and lowered the surface energies of the fibers, thereby increasing its wettability and dispersion within the matrix. On the other hand, PLA-*g*-St/MAH had functional groups with high polarity and reactivity, which were able to be chemically bonded to the surface of the wood flour by an ester bond on one side (a possible reaction mechanism is shown in Figure 6) and combined with the PLA matrix by physical entanglement on another side [44]. In addition, MAH groups in the graft chains of the compatibilizers might also be able to react with the carboxyl groups on the PLA surface, improving the interfacial adhesion between the PLA and the wood flour. Therefore, the stress could be effectively transferred from the wood fiber to the PLA matrix, improving the mechanical properties of the wood flour/PLA composites.

However, excessive PLA-*g*-St/MAH tends to decrease the performance of composites. The mechanical properties of the wood flour/PLA composite decreased when the content of PLA-*g*-St/MAH exceeded 3 wt %, which is in accordance with the rheological behavior of the composite described above. Zhu et al. [26] indicated that only a certain concentration of graft copolymers is required to saturate the interface and produce optimum compatibilization in reactive blending. The suitability of the amount of compatibilizer is relevant to its degree of coverage on the surface of the wood flour particles. If the amount of compatibilizer is extremely low, forming a good coupling molecular layer to achieve the ideal compatibilized effect is difficult, because of the incomplete surface of wood flour particles. When excess PLA-*g*-St/MAH was added, a large amount of compatibilizer migrated around the wood flour surface, and multilayers of macromolecules located at the interface between the wood flour and the PLA matrix were able to form, causing self-entanglement among the PLA-*g*-St/MAH, rather than the PLA matrix. As a result, slippage of wood fibers within the PLA matrix could take place [45,46], which decreased the interfacial properties of the wood flour/PLA composite. Excessive PLA-*g*-St/MAH could cause more side effects and degradation during the process [33], which could also weaken the interaction between the wood flour and the PLA matrix, thus affecting the mechanical properties of the wood flour/PLA composites.

Table 3 shows the effects of grafting degree of PLA-*g*-MAH on the mechanical properties of wood flour/PLA composites. It can be noted that the mechanical properties of the wood flour/PLA composite with PLA-*g*-St/MAH are higher than those of the composite with PLA-*g*-MAH as a compatibilizer. Similar results have been reported previously by Zhu et al. [26] and Gao et al. [33]. This is because the grafting degree of PLA-*g*-St/MAH increases with St as a comonomer. With more MAH grafted onto the PLA, the compatibilizing reaction between PLA-*g*-St/MAH and wood flour became more intense at the interfaces, thus achieving better interfacial adhesion [26], resulting in higher mechanical properties.

### 3.5. SEM

**Figure 7:**
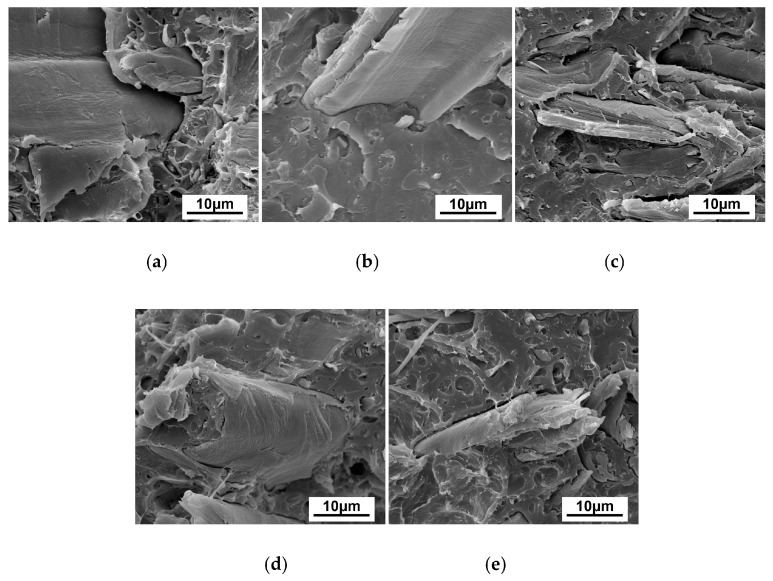
SEM micrographs of the fractured surface of wood flour/PLA composites with different PLA-*g*-St/MAH loading rates. (**a**) WP; (**b**) WPS1; (**c**) WPS2; (**d**) WPS3; (**e**) WPS4.

The morphologies of wood flour/PLA composites with different PLA-*g*-St/MAH loading rates are shown in Figure 7. Without adding PLA-*g*-St/MAH (Figure 7a), the wood flour granules were unevenly distributed, and some holes left by wood fibers pulling out from the PLA matrix could be clearly observed, implying the occurrence of phase separation and the weak interfacial adhesion between the wood flour and PLA. The fracture surface of the wood flour/PLA composite was rough and the interface between the wood flour and the PLA matrix was obvious, indicating poor compatibility between the polar wood flour and the less-polar PLA matrix.

As shown in Figure 7b–e, with the addition of PLA-*g*-St/MAH, the wood flour was wrapped up by the PLA matrix, and the interface between the wood flour and PLA became vague. Some interfaces remained visible in the micrographs of the composites with PLA-*g*-St/MAH; however, they were considerably narrower and fewer than those of the wood flour/PLA composite without PLA-*g*-St/MAH, implying that the PLA-*g*-St/MAH did have an improved interfacial adhesion effect on the wood flour/PLA composites.

With the addition of 3 wt % PLA-*g*-St/MAH (Figure 7d), wood flour was more uniformly dispersed in the PLA matrix, and the occurrence of interfacial debonding phenomena was not as obvious. These results could be attributable to having a suitable PLA-*g*-St/MAH loading rate. Anhydride groups on one end of PLA-*g*-St/MAH chains reacted with the hydroxyl groups on the surface of wood flour by esterification. The other end of the PLA-*g*-St/MAH could also combine with the less-polar PLA matrix by physical entanglement, indicating that the compatibility between the wood flour and the PLA matrix had been improved effectively. Therefore, the loading rate of the PLA-*g*-St/MAH played an important role in the improvement of the interfacial adhesion between the wood flour and the PLA matrix. This phenomenon was consistent with the aforementioned rheological and mechanical properties.

## 4. Conclusions

Styrene-assisted free-radical melt grafting of MAH onto PLA (PLA-*g*-St/MAH) was achieved. PLA-*g*-St/MAH was used as an efficient compatibilizer for wood flour/PLA bio-composites. The effects of the loading rate of PLA-*g*-St/MAH on rheological and mechanical properties, as well as the morphology of the composites, were investigated. Wood flour/PLA composites using PLA-*g*-St/MAH as a compatibilizer exhibited higher storage modulus, complex viscosity, equilibrium torque, and shear heat, indicating that better compatibility was achieved with the addition of PLA-*g*-St/MAH. When the content of PLA-*g*-St/MAH was 3 wt %, the mechanical properties of the composite reached their maximum values, and were higher than those for the composite with 3 wt % PLA-*g*-MAH. The maximum values included increases of 63.06, 15.09, 19.02 and 64.00% in tensile strength, flexural strength, impact strength, and elongation at break, respectively, compared with the uncompatibilized composites. Compatibility of the wood flour/PLA composites was improved significantly after the addition of PLA-*g*-St/MAH because of the good interfacial adhesion between the wood flour and the PLA matrix. PLA-*g*-St/MAH proved to be an effective compatibilizer in wood flour/PLA bio-composites, and a suitable content of PLA-*g*-St/MAH could optimize the mechanical properties of wood flour/PLA bio-composites.

## Figures and Tables

**Table 1:** Compositions of various wood flour/PLA composites.

Sample	Wood flour (wt %)	PLA(wt %)	Relative to the weight of wood flour and PLA	
			PLA-*g*-St/MAH (wt %)	PE wax (wt %)
WP	30	70	0	0.5
WPS1	30	70	1	0.5
WPS2	30	70	2	0.5
WPS3	30	70	3	0.5
WPS4	30	70	4	0.5

Note: the formulation in Table 1 is expressed by weight parts.

**Table 2:** Torque rheological parameters of the wood flour/PLA bio-composites with different PLA-*g*-St/MAH loading rates.

Sample	*T*_e_ (N·m)	Δ*T* (°C)
WP	2.7	8.40
WPS1	3.9	9.89
WPS2	4.0	10.10
WPS3	4.3	11.51
WPS4	3.9	10.60

Note: *T*_e_ represents equilibrium torque; Δ*T* represents shear heat.

**Table 3:** Mechanical properties of wood flour/PLA composites comprising compatibilizer with different grafting degrees.

Sample	Tensile strength (MPa)	Flexural strength (MPa)	Impact strength (kJ·m^−2^)	Elongation at break (%)
WP	30.29 ± 1.71	77.48 ± 3.41	7.78 ± 1.92	1.75 ± 0.23
WPM3	45.93 ± 1.30	86.21 ± 2.97	8.11 ± 0.99	2.69 ± 0.17
WPS3	49.39 ± 0.12	89.17 ± 1.84	9.26 ± 1.49	2.87 ± 0.06

Note: WPM3 represents the wood flour/PLA composite with 3 wt % PLA-*g*-MAH as compatibilizer, PLA-*g*-MAH is maleic anhydride grafted poly(lactic acid) synthesized without styrene; the grafting degree of PLA-*g*-MAH is 0.24%, the grafting degree of PLA-*g*-St/MAH is 0.56%, conducted by titration method [36].
